# Anesthetic Management of a Down Syndrome Patient for Ventricular Septal Defect Repair: A Case Report

**DOI:** 10.7759/cureus.34132

**Published:** 2023-01-24

**Authors:** Haneesha Movva, Karuna Taksande

**Affiliations:** 1 Department of Anaesthesiology, Jawaharlal Nehru Medical College, Datta Meghe Institute of Higher Education and Research, Wardha, IND

**Keywords:** down syndrome, cardiopulmonary bypass, cardiac surgery, asd, vsd

## Abstract

It is commonly known that Down syndrome (DS) and congenital cardiovascular abnormalities go hand in hand. Most frequently, complete atrioventricular septal abnormalities have been linked to DS. Along with DS, ventricular septal defect (VSD), atrial septal defect, tetralogy of Fallot, and patent ductus arteriosus have also been reported. We present a case of DS with VSD who underwent VSD correction. Echocardiography prompted the diagnosis, which was then confirmed by surgery. The patient was successfully transferred out of the hospital. After correcting the VSD, the survival and quality of life of the DS patient have improved.

## Introduction

The range of congenital cardiac defects in Down syndrome (DS) patients varies. A heart abnormality affects roughly 30% of babies with trisomy 21 who also have cardiac malformation. Congenital heart disease, also known as a congenital heart defect, refers to a several anatomical issues with the heart or its major blood arteries that exist from birth. About 14-16% of all congenital cardiovascular problems are ventricular septal defects (VSDs). Congenital cardiovascular defects, the most typical birth defect, affect around eight of every 100 newborns per year, or 1% of live births. It is the leading cause of birth defect-related mortality in the first year of life. There are many clinical signs and symptoms of DS, and they can affect every system of the body. The most serious ones include congenital vascular malformation, low stature, heart disease, gastric illness, orthopedic deformities, and intellectual disability. Undoubtedly, heart disease is the primary cause of good or bad outcomes in these children. Pulmonary arterial hypertension (PAH) may manifest sooner and progress more violently in patients with DS. There are few effective treatments for Eisenmenger syndrome, such as the use of vasodilators and systemic arterial to pulmonary shunts surgically placed to increase oxygen saturation, and the chance for fatality is exceptionally high in this group of patients. The three main fatalities in patients with DS and cardiac illness are cardiac arrest, infections, and pulmonary edema. This last step causes a survival rate drop of up to 58% and manifests early in individuals with atrioventricular septal defect (AVSD). In DS, upper airway obstruction is expected to occur 30-50% of the time. Individuals with DS have an increased risk of infection, particularly respiratory tract infections, which are more prevalent in this group. A prevalent cause of death, bronchopneumonia, has a mortality rate 124 times higher than the overall population [1]. Here, we discuss the case of a patient with this condition who had a VSD [2].

## Case presentation

**Figure 1:**
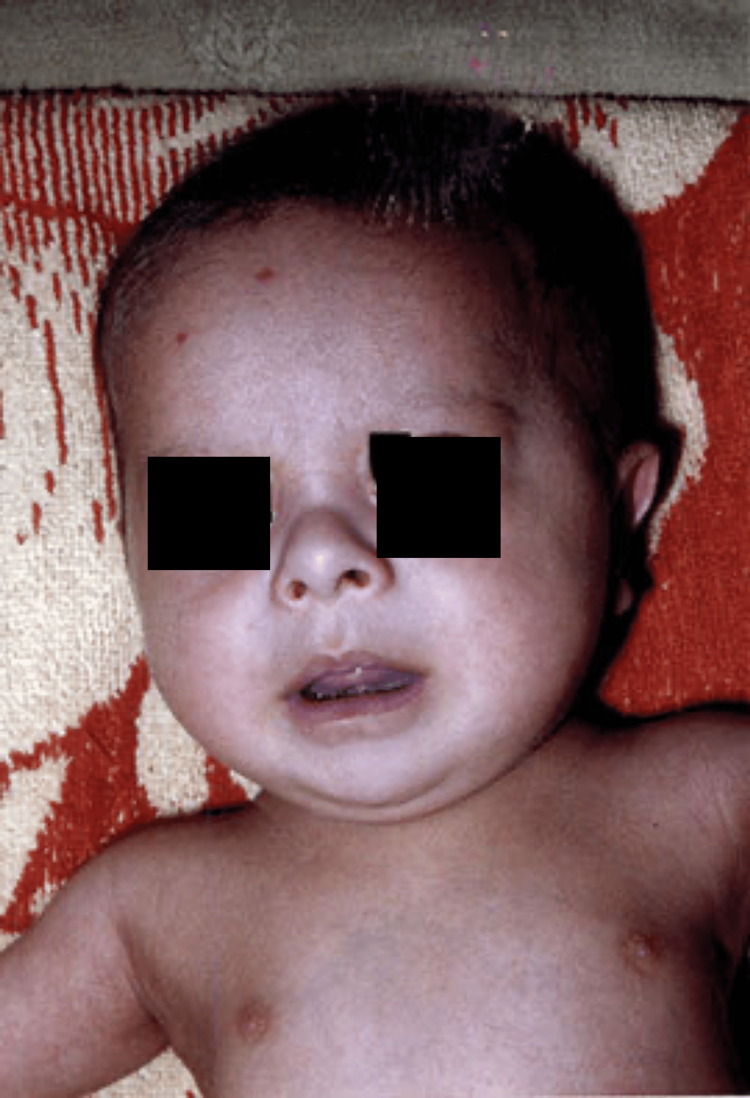
The depressed nose.

A four-month-old hospitalized boy showed facial dysmorphism, upward slanting of the palpebral fissure, a flat nasal bridge, and a protruding tongue. He also had small, wide hands and a flat occiput which are some of the signs of DS (Figure 1).

**Figure 2:**
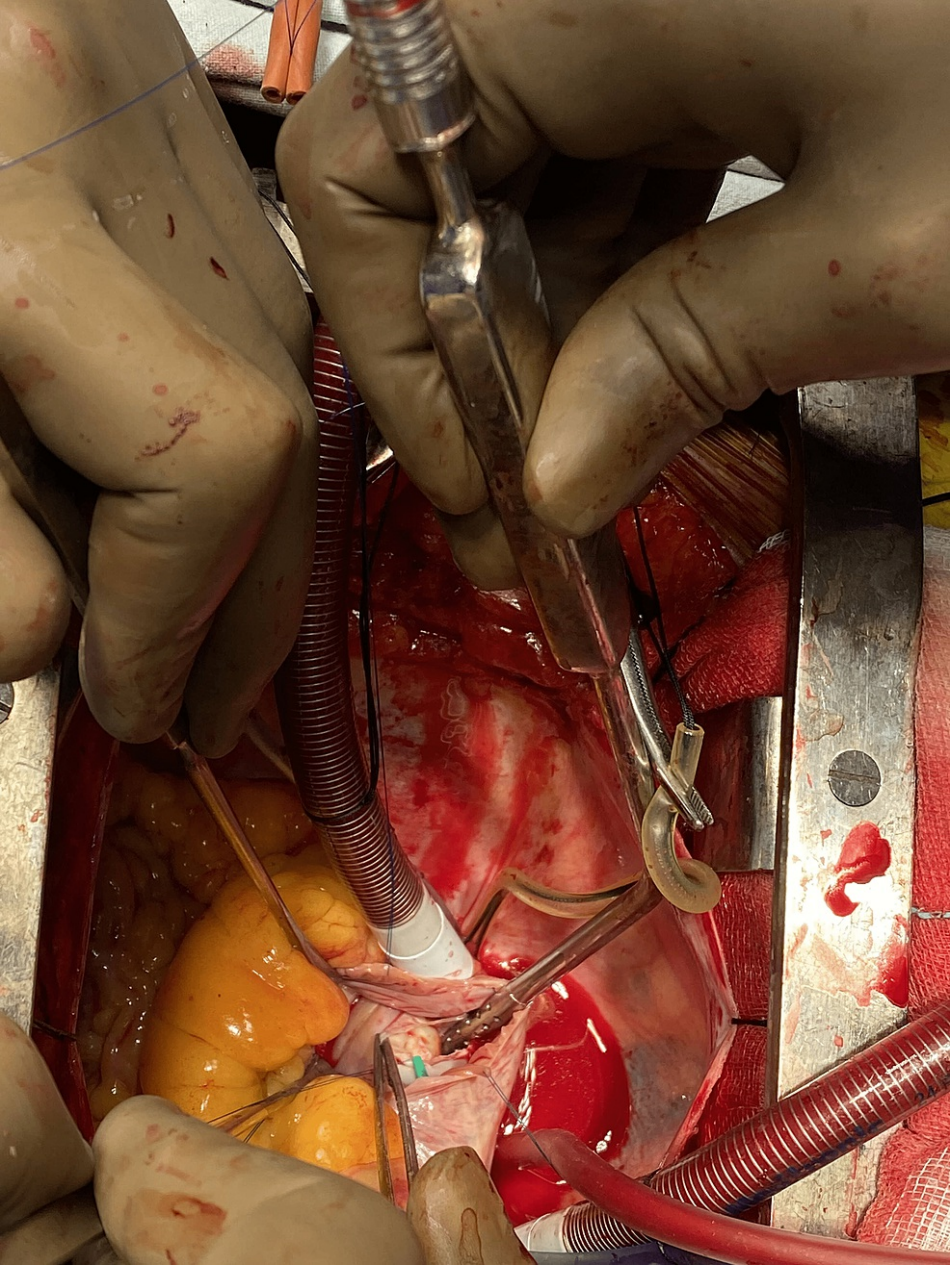
The polytetrafluoroethylene patch closing the defect.

The chest could be auscultated for vesicular breath sounds. During the cardiovascular examination, a pan-systolic murmur was heard that was most audible in the left fourth intercostal space in the parasternal region and resonating through the precordium, supporting the clinical diagnosis of VSD. Other systems were unremarkable on examination. An echocardiography study revealed a moderately sized peri-membrane VSD with a left-to-right inlet extension, small atrial septal defect (ASD), left-to-right shunt, dilated left atrium (LA) and left ventricle (LV), and severe PAH. The renal function test, liver function test, and complete blood count were all within normal ranges. On the day of the surgery, after written informed consent from the parent and postoperative intensive care unit (ICU) stay, the patient was wheeled to the operating room and placed under the inhalational agent sevoflurane, with SpO_2_ monitoring. Next, a 24 g intravenous (IV) line was secured, the patient was connected to an electrocardiogram (ECG), and was induced using an injection of midazolam 0.05 mg/kg, fentanyl 2 µg/kg, and vecuronium 0.1 mg/kg. The airway was then secured with 4.0 uncuffed endotracheal tube after bilateral equal air entry. Ventilation was maintained at a rate of 32 breaths per minute, a total volume of 20 mL, an I:E ratio of 1:2, and FiO_2_ of 60%. Vecuronium top-ups and sevoflurane were used to maintain the patient. For invasive blood pressure monitoring, femoral arterial catheterization was performed. Heparin 300 IU was administered intravenously after the sternotomy. The clamp on the venous line was released, and cardiopulmonary bypass (CPB) was started when the activated clotting time was more than 420 seconds. Sevoflurane, IV fluids, and ventilation were stopped after appropriate flows, and venous drainage was in place. To stop movement, muscle relaxants were provided again. Anesthesia was maintained by utilizing IV or inhalational medications delivered via a vaporizer in the circuit’s fresh gas line. VSD was sealed using a polytetrafluoroethylene patch (Figure 2).

For the discontinuation of CPB, the arterial blood was warmed to 32°C. Arterial blood gas was taken during rewarming for blood gas analysis. Injection protamine ensured enough anticoagulation during rewarming, and dissociation from CPB and activated clotting time confirmed it. Maintaining sufficient analgesia, amnesia, and neuromuscular blockade are significant anesthetic concerns during rewarming. It is possible to provide more opioids, benzodiazepines, and relaxants. Nitroglycerin pop-ups are given to regulate blood pressure and aid in rewarming if the mean arterial pressure is raised. To prevent air embolism into the cerebral or coronary circulations following operations where the heart was opened, de-airing maneuvers were performed under transesophageal echocardiography supervision. The venous return lines will be clamped in conjunction with positive-pressure ventilation to force air from the pulmonary veins forward. The removal of the aortic cross-clamp restores coronary perfusion. Once the skin closure was begun, injection Neomol 15 mg/kg IV was given with injection Emeset 0.1 mg/kg. Once the surgery was done and the patient was on adequate spontaneous ventilation, the reversal was achieved with injection myopyrolate (0.05 mg/kg) injected slowly. The patient was fully awake, conscious, extubated, and shifted to the ICU.

## Discussion

DS, or trisomy 21, was first identified in 1866 and is a genetic disorder. Overall, 95% of cases are primary trisomies, while the remaining 5% are translocation and mosaic variants. One in 650 live births is the frequency of presentation. About 1% of the population experiences this frequency. Cardiac abnormality is the leading cause of mortality during the first two years of life. The survival chances of DS patients are 72% lower due to congenital cardiac abnormalities. Higher success rates than AVSD are seen in the early stages of ASD and VSD. Nevertheless, research from the last 20 years revealed that survival rates for newborns with DS and a healthy heart improved from 93% to 97% and from 78% to 90%, respectively. The pulmonary vascular resistance of children with congenital cardiac disease and DS is exceptionally high, and they are more likely to experience early pulmonary vascular bed injury. Atrioventricular valve regurgitation and the evolution of pulmonary vascular disease make the first six months of life the optimal period for some repairs. Some DS patients have undergone successful surgeries as late as their second year, while others succumb to a pulmonary hypertensive crisis as early as in the first six months [3]. Anesthetic considerations of DS are microcephaly with a large tongue. Hence, there are chances of difficult intubation with the possibility of subglottic stenosis, so a small endotracheal tube has been considered. Atlanto-oocipital instability also poses a problem during laryngoscopy. The hemodynamic relevance of VSDs is influenced by the size of the VSD, the pressure in the right and left ventricular chambers, and pulmonary resistance. Due to the almost equal pressures in the right and left ventricles and the absence of shunting, a VSD may not be visible at birth. These abnormalities manifest clinically when the shunt increases and increases the pressure differential between the ventricles [4]. The size of the shunt in a VSD is significantly influenced by the severity of the lesion and pulmonary vascular resistance. In the absence of pulmonary hypertension or obstruction to the right ventricle, the direction of the shunt is left to right, with similar volume overload in the left atrium, left ventricle, and pulmonary artery [5]. When there is a right ventricular block caused by muscular bundles, pulmonary stenosis, or higher pulmonary vascular resistance, the volume of the shunt is restricted, and depending on the pressure gradient, it may be right to left. Eisenmenger syndrome is brought on by continuous left-to-right shunting, frequently at higher shunt flow rates. The high pulmonary artery pressure causes the ventricular level shunt to reverse, hypoxemia, cyanosis, and secondary erythrocytosis, all of which are irreversible [6]. The occlusion of muscles can cause muscular VSDs to spontaneously close. The growth of tricuspid valve aneurysms can seal peri-membranous defects. The prolapse of the right aortic cusp might close infundibular abnormalities. Any of these methods can cause a defect to shrink, which alters the lesion’s hemodynamic importance [7].

## Conclusions

Improvements in critical care facilities, the use of vasodilators, and successful management of early congenital heart defect repair to prevent sepsis, prognosis, and anesthetic considerations were successfully managed, all of which contributed to the patient’s ability to live longer with a higher quality of life
